# Smartphone-Based Acoustic Sensing for Breathing and Heartbeat Detection via AoA Clustering in Indoor Environments

**DOI:** 10.3390/s26144591

**Published:** 2026-07-20

**Authors:** Kounkou Vincent, Ijaz Khan, Ke Sun, Yizhi Shao, Zhantu Liang, Asif Ullah, Tao Gong

**Affiliations:** 1 Institute of Ultrasonic Technology, Shenzhen Polytechnic University, Shenzhen 518055, China; ladis_vincent@szpu.edu.cn (K.V.); khanijaz@stu.hit.edu.cn (I.K.); sunke0101@szpu.edu.cn (K.S.); yzshao@szpu.edu.cn (Y.S.); liangzhantu@szpu.edu.cn (Z.L.); 2 Shenzhen Institutes of Advanced Technology, Chinese Academy of Sciences, Shenzhen 518055, China; 3 Institute of Intelligent Manufacturing Technology, Shenzhen Polytechnic University, Shenzhen 518055, China

**Keywords:** acoustic signals, smartphone, AoA, BR, HR

## Abstract

Smartphones incorporate acoustic components, including a speaker and multiple microphones, which can be used as a low-cost, contactless platform for vital signs monitoring. However, extracting breathing rate (BR) and heart rate (HR) from smartphone acoustic reflections remains challenging in indoor environments because thoracic reflections are weak and are often mixed with static clutter, hand motion, environmental multipath, and other dynamic sources. In this work, we present a smartphone-based frequency-modulated continuous wave (FMCW) acoustic sensing system that enables simultaneous BR and HR estimation using the integrated speaker and two physical microphones. Instead of processing the received signal as a single, mixed signal, the proposed method leverages distance information from the FMCW beat frequency and an angular phase index (AoA information), derived from dual-microphone and virtual aperture processing, to organize moving reflectors into a joint distance–angle–time representation. A 3D-DBSCAN clustering module is then applied to this representation to separate candidate dynamic sources from static and multipath components, without presupposing the number of sources. To further handle ambiguous cases where multiple candidate dynamic sources are detected, a Siamese similarity network is introduced as a conditional second-stage source-association module. The Siamese model compares candidate thoracic waveforms and estimates whether multiple detected components are likely to originate from the same physical source or different sources, thus improving source selection without resorting to classical blind source separation. The system was evaluated on 20 participants in two indoor environments, a laboratory and a bedroom, using three consumer smartphones and an electrocardiogram (ECG) reference device. In the smartphone-only blind configuration, the proposed pipeline achieved MAEs of 2.312 bpm for HR and 1.394 bpm for BR. In the ECG-assisted calibrated configuration, which is used to evaluate physiological coherence rather than deployable smartphone-only performance, the errors decreased to 0.462 bpm for HR and 0.091 bpm for BR. These results demonstrate that spatial clustering and conditional Siamese source pairing improve the robustness of acoustic vital sign detection using smartphones in indoor environments.

## 1. Introduction

Contactless monitoring of breathing rate (BR) and heart rate (HR) has become an important research topic for mobile health, sleep monitoring, human–computer interaction, and unobtrusive physiological assessment. Recent advances in acoustic signal processing have enabled a wide range of sensing applications, including ultrasonic sensing, respiratory monitoring, and non-contact physiological measurement [1,2]. Compared to wearable devices, smartphone acoustic sensing is attractive because modern smartphones already integrate a speaker and multiple microphones, thus enabling active sensing without additional hardware. By emitting high-frequency acoustic signals and analyzing echoes reflected by the human body, a smartphone can potentially record rib cage movements induced by respiration and heartbeats.

Despite this potential, reliable extraction of vital signs from the acoustic signals of commodity smartphones remains challenging in realistic indoor environments. The reflected physiological signal is weak, non-stationary, and often disturbed by static interference, hand movements, multipath propagation, and other dynamic reflectors. Existing approaches in machine learning [3], deep learning [4], and conventional signal processing [5] have demonstrated effectiveness in various detection tasks. Classical spectral methods, such as the fast Fourier transform (FFT) and short-term Fourier transform (STFT) analysis, remain widely used to extract the dominant periodic components of acoustic or radio frequency (RF) signals [6]. Similar challenges have also been investigated using sensing systems based on mmWave [7,8,9], LoRa [10], and UWB [11,12,13], where range, Doppler, or phase information is used to distinguish human movement from background interference. These systems have been applied to the simultaneous monitoring of vital signs of multiple people [14], hand-gesture sensing and gesture recognition during handwriting [15,16], and multi-source audio separation [17]. However, most acoustic vital sign monitoring systems for smartphones still rely primarily on temporal or spectral information and do not explicitly exploit spatial cues to resolve source ambiguity.

A major limitation of non-spatial acoustic vital sign detection lies in the typical processing of the received signal as a single mixed signal. In an indoor room, a smartphone receives not only direct reflections from the chest but also indirect reflections from walls, desks, beds, clothing, hands, and other moving objects. When these components overlap in the respiratory or cardiac frequency bands, a purely spectral method risks selecting a strong but non-thoracic reflection, thus leading to an erroneous estimation of the respiratory or cardiac rate. This problem is particularly critical for heartbeat detection, as the chest wall displacement induced by the heartbeat is much smaller than the respiratory displacement and can be easily masked by stronger, non-physiological components. Therefore, reliable acoustic detection using a smartphone requires not only filtering but also explicit identification and source association.

Deep learning has been extensively explored for the separation of acoustic sources. Lluís et al. [18] showed that waveform-domain separation overcomes some limitations of spectrogram-based reconstruction, while Grais et al. [19] demonstrated the potential of audio separation based on a multichannel autoencoder. These works highlight two important observations for our problem. First, phase information is essential for reconstructing non-stationary acoustic components. Second, manually defined spatial features, such as level or phase differences between channels, may not fully capture spectral, temporal, and spatial characteristics simultaneously. Nevertheless, directly applying end-to-end deep separation methods to smartphone acoustic vital sign sensing remains challenging because large labeled datasets are difficult to collect, the physiological signal is weak, and black-box separation does not explicitly preserve the physical relation between FMCW beat frequency, range, phase, and chest-wall displacement.

The physiological objective of this work is to extract dynamic components related to breathing rate, heart rate, and subtle body movements [20,21,22]. Although convolutional neural networks (CNNs) [23], LSTM-based models [24], and transformer-based methods [25] have been successfully applied to the analysis of physiological and acoustic signals, their direct application to raw smartphone acoustic signals is insufficient in our context. In particular, the weak cardiac component is easily masked by respiration, multipath artifacts, and motion artifacts. Therefore, a physically interpretable preprocessing strategy is necessary before applying a learning-based association.

To solve this problem, we formulate the extraction of acoustic vital signs from a smartphone as a spatially constrained source selection problem, rather than a simple signal denoising problem. The smartphone’s speaker emits an FMCW acoustic chirp, and the two integrated microphones receive the reflected echoes. The beat frequency provides range information related to round-trip delay, while the phase relationship between the microphone channels and the slow-time FMCW structure provides a phase-based angular index [26]. Advanced AoA estimation techniques have been widely investigated in array signal processing, including maximum-likelihood AoA estimation using orthogonal projections [27]. These methods are designed for calibrated array configurations and can provide high-resolution angular estimates under appropriate array assumptions. Since a smartphone has only two physical microphones and the spacing between these microphones is greater than half the acoustic wavelength at 18 kHz, the angular information is not treated as a perfectly calibrated absolute angle of arrival. Rather, it is used as a convenient angular index to organize coherent dynamic reflections into a joint distance/range-angle-time representation.

The main technical novelty of this work does not lie in the isolated use of FMCW, MTI filtering, 3D-DBSCAN, Wiener filtering, VMD, or Siamese learning techniques, as each of these methods is known individually. Rather, the novelty lies in the integration of these components within a physically guided and conditional source association framework for acoustic detection of vital signs using a smartphone, which we call the “divide-and-conquer” method. More precisely, the proposed system first transforms the two-microphone FMCW signal into a range-angle-time map. A 3D-DBSCAN module is then applied to this representation to discover candidate sources without presuming a specific number of reflectors. This allows the system to separate static clutter, background noise, direct thoracic reflections, and any indirect dynamic reflections before estimating BR and HR. After clustering, each candidate is evaluated using a physiological spectral score, adaptive Wiener filtering, and a VMD-based decomposition. Finally, a Siamese similarity model is applied only when multiple dynamic candidates are detected. Therefore, the Siamese network is not used as a generic classifier for each recording, but rather as a second-stage conditional ambiguity resolver that compares candidate waveforms and estimates whether multiple detected components are likely to originate from the same physical source or from different sources.

This design differs from classical blind source separation approaches, such as independent component analysis (ICA) and FastICA [28,29]. Instead of assuming statistical independence between unknown mixed components, the proposed method takes advantage of the physical structure of FMCW detection and the spatial coherence of the acoustic reflections. The 3D-DBSCAN module performs geometry-aware candidate discovery, while the Siamese module performs waveform-level source association in ambiguous multi-candidate cases. This combination is particularly useful in realistic indoor environments, where multipath reflections may exhibit similar physiological frequency content but different range-angle-time behavior.

The proposed system was evaluated in a laboratory and a bedroom using smartphone-only acoustic recordings and an ECG reference device. The evaluation distinguishes between two configurations. In the “blind” configuration (smartphone-only), the ECG reference is used solely to calculate the final error. In the “calibrated with ECG” configuration, the time and frequency information from the ECG is also used for source association and peak-level calibration. This distinction is important because the calibrated configuration demonstrates the physiological coherence of the selected acoustic source, whereas the “blind” configuration reflects the independent smartphone-only estimation performance.

The main contributions of this work are summarized as follows:

- We propose a smartphone-based FMCW acoustic detection framework for the simultaneous estimation of BR and HR, using only the built-in speaker and two physical microphones. Unlike methods that treat the received echo as a single mixed waveform, the proposed system organizes the reflections into a joint range-angle-time representation.
- We present a source detection module based on the 3D-DBSCAN technique, which clusters acoustic reflections according to their range, slow time, and a phase-based angular index (cue). This module does not assume a predefined number of sources and is designed to separate thoracic candidates from static clutter and multipath components in indoor environments.
- We integrate a conditional Siamese source-association step as an auxiliary ambiguity-resolution module for recordings in which multiple dynamic acoustic candidates are detected. The current Siamese validation is performed at the candidate-pair level and is therefore interpreted as within-cohort candidate-association validation rather than as evidence of subject-independent generalization.
- We combine physiological scoring, adaptive Wiener filtering, and VMD-based decomposition to extract respiratory and cardiac components from the selected thoracic candidate. The method explicitly preserves the distinction between the estimation of the respiratory band for BR and the estimation of the cardiac band for HR.
- We provide an evaluation in a blind smartphone-only setting and an ECG-assisted calibrated setting, clarifying the difference between independent acoustic estimation and ECG-assisted source association. This separation improves the transparency of the reported results.

## 2. Related Work

### 2.1. Vital Sign Detection Using Smartphone Cameras

Previous studies have also prospectively validated smartphone-based algorithms for heart-rate and respiratory-rate measurement [30]. Methods for extracting vital signs from smartphones rely primarily on remote photoplethysmography (rPPG), which measures subtle variations in light absorption due to pulsations of blood volume in microvascular tissues of the face. Ref. [31] demonstrated the viability of using front-facing smartphone cameras to capture these signals, allowing the extraction of HR, oxygen saturation (SpO_2_), and BR through advanced signal filtering and amplification techniques.

Furthermore, Ref. [32] explored non-contact neck imaging using a standard smartphone camera and ambient lighting, achieving correlation coefficients (r) of 0.98 for HR and 0.85 for BR through video analysis. Despite their accessibility, camera-based systems are highly susceptible to motion artifacts and require optimal lighting conditions, which limits their widespread deployment in real-world environments.

### 2.2. Acoustic Detection of Vital Signs Using Smartphones

Acoustic detection has emerged as a complementary modality, using smartphone speakers and microphones to capture chest vibrations induced by cardio-respiratory activity. Acoustic cardiography (ACG), one of the first studies in this field, used FMCW to extract phase variations induced by heartbeats [33]. To date, most acoustic systems have focused primarily on respiratory rate due to its higher SNR.

For respiratory monitoring, AcHand [34] achieves a mean absolute error of 0.341 bpm by tracking subtle cardiac movements, but remains susceptible to artifacts related to hand movements. AcBreath [35] uses bimodal acoustic signals and AcBreathNet to reconstruct respiratory signals, achieving an overall error rate of 0.275 bpm. Thus, for HR monitoring, LoEar [36] extends the detection range to 6.5 m, but still relies on a single microphone and adaptive filtering to extract cardiac signals from breathing-dominated channel frequency response (CFR) measurements. More recently, FreeHold [37] represents a significant advance in HR detection, with an error rate of 0.364 bpm and robust beat-by-beat tracking, thanks to a contrastive encoder-discriminator learning model.

Although LoEar and FreeHold demonstrate the potential for long-range or high-precision HR monitoring, they treat vital signs as a single aggregated signal, typically combining data from all reflective surfaces. To our knowledge, no previous work has combined FMCW-based telemetry, AoA estimation, and spatial clustering specifically for the simultaneous detection of respiration and heartbeats from smartphone acoustic signals. Existing methods therefore fail to isolate the unique reflective signature of the chest from multipath interference, a crucial step in capturing the nuanced movements of the heart, which are an order of magnitude smaller than respiratory motion.

## 3. Method

### 3.1. System Overview

**Figure 1:**
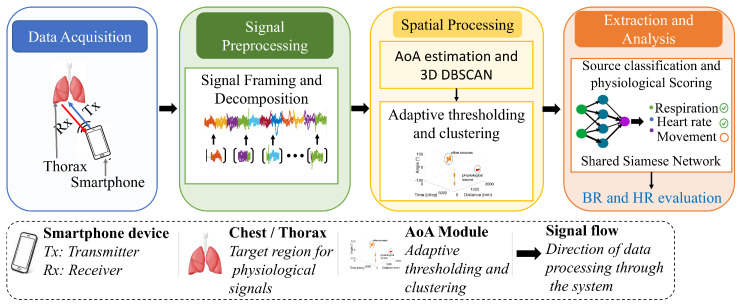
Architecture of the proposed framework.

We propose a divide-and-conquer framework that decomposes smartphone acoustic vital sign sensing into a sequence of physically interpretable processing modules. Figure 1 illustrates the overall architecture of the proposed method. First, data acquisition is performed by transmitting an FMCW acoustic chirp from the smartphone loudspeaker and recording the reflected echoes using the two built-in microphones. The reflected signals are modulated by small thoracic motions induced by respiration and heartbeat, but they also contain static clutter, environmental multipath, and non-physiological dynamic interference.

The acquired two-channel acoustic signal is segmented into FMCW chirps and then converted into a time–distance representation using an FFT. A moving target indicator (MTI) filter is applied to the slow-time dimension to suppress static reflections from walls, furniture, and other stationary objects while preserving the dynamic components related to body movement. Spatial processing is then performed using an angular phase index obtained through two-microphone/virtual aperture processing. The resulting information is organized into a joint range-angle-time space. Adaptive thresholding, followed by 3D-DBSCAN clustering, enables the detection of potential reflection sources without presupposing a specific number of sources. After clustering, each candidate source is converted into a displacement signal from the phase of the corresponding range bin. The candidates are then evaluated using physiological scoring based on their distance, amplitude, angular consistency, respiratory-band energy, and cardiac-band energy. The candidate with the most plausible thoracic signature is selected for subsequent physiological extraction. Adaptive Wiener filtering is applied to reduce residual interference, and VMD-based decomposition is used to separate respiratory and cardiac components before estimating BR and HR.

In addition to this classical source selection method, the framework includes a conditional Siamese metric learning step for ambiguous recordings. The Siamese model is trained from candidate acoustic windows extracted after the first processing pass. Each candidate displacement signal is divided into fixed-length normalized windows, and positive and negative pairs are constructed from thoracic and non-thoracic candidate windows. The network uses two weight-sharing one-dimensional CNN branches to map each input window into an embedding vector, and it is trained with a contrastive loss so that windows from similar thoracic sources are mapped closer than windows from different or interfering sources. During inference, the Siamese stage is not applied blindly to every recording. It is activated only when more than one dynamic candidate is available, because this is the case where source ambiguity exists. If only one dynamic source is detected, the final score is computed from the physiological and ECG-consistency scores without adding a Siamese similarity term. When several dynamic candidates are present, the trained Siamese model computes pairwise similarity between candidate displacement signals and compares each candidate with a reference bank of thoracic-like windows. These similarity scores are then combined with the candidate’s base physiological score to refine the final thoracic source selection. This conditional use of Siamese learning avoids unnecessary neural scoring in simple single-source recordings while improving source association in multipath or multi-candidate cases.

### 3.2. FMCW Acoustic Signal Model and Data Acquisition

The smartphone loudspeaker emits an FMCW acoustic chirp with sweep duration $T_{c}$ and bandwidth *B*. In our implementation, the sampling frequency is $F_{s}=48$ kHz, the carrier frequency is $f_{c}=18$ kHz, the bandwidth is B=2 kHz, and each sweep contains M=512 samples. Therefore, the sweep duration is as follows:

(1) $$T_{c}=\frac{M}{F_{s}}=10.67\;\mathrm{ms}.$$

**Figure 2:**
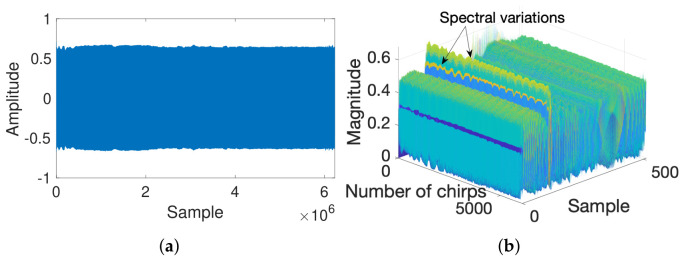
(**a**) Raw acoustic signal in the time domain; (**b**) Corresponding time frequency representation.

The mathematical expression for the transmitted acoustic chirp is written as follows:

(2) $$s_{\mathrm{tx}}\left(t\right)=A\cos\left(2\pi f_{c}t+\pi \frac{B}{T_{c}}t^{2}\right),\;0\leq t\leq T_{c},$$

where *A* is the signal amplitude and $\mu =\frac{B}{T_{c}}$ is the slope of the chirp. A target located at a distance *R* produces a delayed echo represented as follows:

(3) $$s_{\mathrm{rx}}\left(t\right)=A_{r}\cos\left(2\pi f_{c}\left(t-\tau \right)+\pi \mu {\left(t-\tau \right)}^{2}\right),$$

where $A_{r}$ is the received amplitude, c=343m/s is the speed of sound, and $\tau =\frac{2R}{c}$ is the round-trip propagation delay. Figure 2a shows the original raw signal captured by the smartphone.

In the recorded smartphone data, the two channels correspond to two real microphone waveforms rather than a direct in-phase/quadrature pair. Therefore, before phase-based processing, each real microphone waveform is converted into an analytic signal using the Hilbert transform. If $x_{1}\left[n\right]$ and $x_{2}\left[n\right]$ denote the two real microphone recordings, their analytic forms are the following:

(4) $$z_{1}\left[n\right]=x_{1}\left[n\right]+jH\left\{x_{1}\left[n\right]\right\},$$

(5) $$z_{2}\left[n\right]=x_{2}\left[n\right]+jH\left\{x_{2}\left[n\right]\right\},$$

where H{·} denotes the Hilbert transform. This clarification is important because the raw data format is a two-microphone real acoustic recording, not a single complex I/Q stream.

After mixing the received signal with the transmitted chirp or equivalently analyzing the beat component of the FMCW sweep, the beat frequency is related to the target distance by the following calculation:

(6) $$f_{b}=\frac{2\mu R}{c}=\frac{2BR}{cT_{c}}.$$

Thus, the target distance can be estimated as follows:

(7) $$R=\frac{cf_{b}}{2\mu }=\frac{cT_{c}f_{b}}{2B}.$$

The resulting complex baseband signal can be determined as follows:

(8) $$Y_{s}\left(t\right)=I\left(t\right)+jQ\left(t\right)=\frac{AA^{\prime }}{2}e^{j\left(2\pi f_{b}t+\phi_{0}\right)},$$

where the initial phase $\phi_{0}=\frac{4\pi f_{c}R}{c}$.

Moreover, $f_{b}$ and $\phi_{0}$ establish the fundamental relationship between the instantaneous frequency (IF) of the received signal and the target distance *R*, as well as between the phase and the sub-wavelength displacement of the target. Figure 2b illustrates the spectrogram after obtaining the baseband signal from the chirp matrix.

### 3.3. Discrete-Time Framing and Range Processing

We now move from the continuous-time signal model to the discrete-time implementation. The analytic microphone signal is sampled at $F_{s}$, and the discrete-time signal is segmented into consecutive FMCW sweeps. Let $M=T_{c}F_{s}$ be the number of samples per sweep and let K=⌊N/M⌋ be the number of complete sweeps in a recording of *N* samples. The sweep matrix is calculated as follows:

(9) $$S_{\mathrm{chirp}}=\left[\begin{array}{cccc} z[0] & z[M] & \cdots & z[M(K-1)]\\ z[1] & z[M+1] & \cdots & z[M(K-1)+1]\\ \vdots & \vdots & \ddots & \vdots \\ z[M-1] & z[2M-1] & \cdots & z[MK-1] \end{array}\right]\in C^{M\times K}.$$

**Figure 3:**
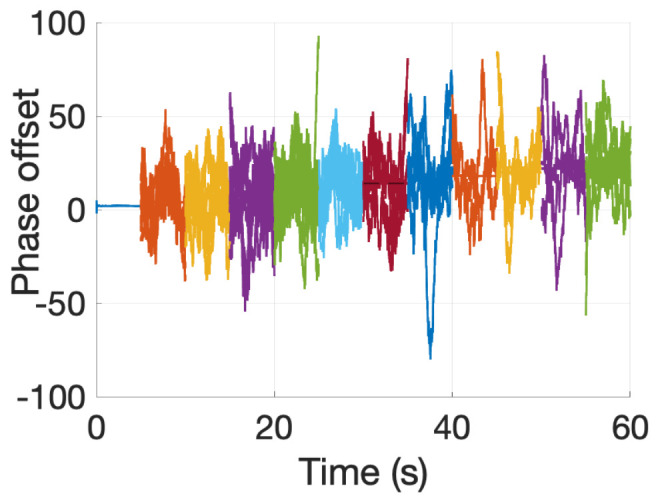
Segmentation of the recorded FMCW signal.

Each column corresponds to one FMCW sweep, while the rows represent the fast-time samples inside a sweep. This organization is essential because it aligns the signal samples that share the same time offset across consecutive chirps, enabling subsequent Fourier analysis along the fast-time axis. Figure 3 shows the framed signal obtained from each chirp round. This discrete notation is used in the rest of the signal-processing pipeline.

### 3.4. Signal Range Estimation Using a Fast-Time FFT

To extract range information, we apply a fast Fourier transform (FFT) along each column of $S_{\mathrm{chirp}}$ (the fast-time dimension). A Hamming window $w\in R^{M}$ is first applied to reduce spectral leakage:

(10) $$S_{w}\left[m,k\right]=S_{\mathrm{chirp}}\left[m,k\right]\;w\left[m\right],$$

where

(11) $$w\left[m\right]=0.54-0.46\cos\left(\frac{2\pi m}{M-1}\right),\;m=0,\ldots ,M-1.$$

The range profile is obtained by applying the fast-time FFT, as follows:

(12) $$R\left[r,k\right]=\sum_{m=0}^{M-1}S_{w}\left[m,k\right]e^{-j2\pi \frac{rm}{M}},\;r=0,\ldots ,M-1.$$

Due to the Hermitian symmetry of the FFT for real-valued inputs, only the first M/2 frequency bins contain unique information. The beat frequency $f_{b}$ is related to the FFT bin index *r* by

(13) $$f_{b}\left(r\right)=\frac{r}{M}F_{s}.$$

Substituting Equation (13) and using $M=T_{c}F_{s}$, we obtain the following:

(14) $$R_{r}=\frac{cT_{c}}{2B}\frac{rF_{s}}{M}=\frac{cr}{2B}.$$

Therefore, the range resolution can be estimated as follows:

(15) $$\Delta R=\frac{c}{2B}.$$

With B=2 kHz, this gives:

(16) $$\Delta R=\frac{343}{2\cdot 2000}=0.08575\;m=8.575\;\mathrm{cm}.$$

Only the first M/2 FFT bins are retained for positive beat frequencies. The theoretical maximum positive range represented by the fast-time FFT is therefore approximately

(17) $$R_{\max}\approx \frac{cM}{4B}.$$

In practice, the physiological analysis is restricted to a near-field region. In the implementation, candidates for physiological reflectors are searched within a maximum range of 150 cm, and thoracic candidates are constrained to the 25–110 cm interval.

### 3.5. Static Clutter Suppression Using MTI Filtering

Indoor acoustic recordings contain strong static reflections of walls, furniture, and other stationary surfaces. These static components can mask weak thoracic reflections. To suppress them, we apply a moving target indicator (MTI) filter along the slow-time dimension. For a fixed range bin *r*, the moving-average clutter estimate is

(18) $$\bar{R}\left[r,k\right]=\frac{1}{W}\sum_{\ell =0}^{W-1}R\left[r,k-\ell \right],$$

where W=50 chirps in the implementation (approximately 0.53 s given $T_{c}=10.67$ ms). This window length is chosen to be long enough to capture static reflections (which remain constant), but short enough to adapt to slow environmental changes. The MTI output is calculated as follows:

(19) $$R_{\mathrm{MTI}}\left[r,k\right]=R\left[r,k\right]-\bar{R}\left[r,k\right].$$

Thus, Equation (13) can be interpreted as a high-pass filter in the slow-time dimension. To see this, consider the temporal response of a static target: its range profile remains constant over *k*, so subtraction eliminates it. In contrast, a moving target exhibits temporal variation, so subtraction does not eliminate its signal. The discrete-time frequency response of the length-*W* moving average is a Dirichlet kernel, which is defined as follows:

(20) $$H_{\mathrm{MA}}\left(e^{j\omega }\right)=\frac{1}{W}e^{-j\omega (W-1)/2}\frac{\sin(W\omega /2)}{\sin(\omega /2)}.$$

Therefore, the MTI high-pass response is as follows:

(21) $$H_{\mathrm{MTI}}\left(e^{j\omega }\right)=1-H_{\mathrm{MA}}\left(e^{j\omega }\right).$$

The normalized digital angular frequency ω is related to the physical slow-time frequency *f* as follows:

(22) $$\omega =2\pi \frac{f}{F_{\mathrm{PRF}}},$$

where $F_{\mathrm{PRF}}=\frac{1}{T_{c}}=93.75\;\mathrm{Hz}$ is the sweep repetition frequency. Since $H_{\mathrm{MTI}}\left(e^{j0}\right)=0$, the filter removes stationary or very slowly varying clutter while preserving dynamic components in the respiratory and cardiac bands. The energy map used for candidate detection is as follows:

(23) $$E\left[r,k\right]={\left|R_{\mathrm{MTI}}\left[r,k\right]\right|}^{2}.$$

This energy map serves as an input for source detection, because higher energy indicates the presence of moving scatterers.

### 3.6. Phase-Based Angular Cue and Virtual Aperture

The two smartphone microphones provide phase information that can be used to obtain a spatial cue. However, because the microphone spacing of a consumer smartphone is much larger than half the acoustic wavelength at 18 kHz, a direct two-microphone phase-difference AoA estimate is spatially ambiguous. For example, at $f_{c}=18$ kHz, $\lambda =\frac{c}{f_{c}}\approx 1.9\;\mathrm{cm}$, while the physical spacing between the top and bottom microphones can be several centimeters. Therefore, the angular estimate used in this work is not interpreted as a calibrated absolute physical AoA. Instead, it is used as a phase-based angular cue to cluster dynamic reflections.

For two received signals with a differential propagation delay Δτ, the phase shift of an FMCW chirp is generally time-dependent:

(24) $$\Delta \phi \left(t\right)\approx 2\pi \left(f_{c}+\mu t\right)\Delta \tau .$$

The simplified narrowband form $\Delta \phi \approx 2\pi f_{\mathrm{eff}}\Delta \tau$ is valid only when the bandwidth is small relative to the carrier and the phase is evaluated locally within a narrow beat-frequency/range bin. In our implementation, the angular feature is therefore treated as a local phase-consistency cue rather than an absolute AoA measurement. The effective frequency $f_{\mathrm{eff}}$ is approximated by the acoustic carrier frequency, and the residual chirp-dependent phase term is absorbed into the empirical angular cue used by the clustering.

To obtain an angular feature for each range–time cell, the implementation uses a slow-time virtual aperture formed by circularly shifting the MTI range profile. This operation should not be interpreted as creating physical microphones. Rather, it produces several delayed slow-time views of the same range profile, which are used to measure phase consistency over time. With $N_{\mathrm{virt}}=4$, the virtual channels are

(25) $$v_{i}\left[r,k\right]=R_{\mathrm{MTI}}\left[r,k+\Delta k_{i}\right],\;i=1,\ldots ,N_{\mathrm{virt}},$$

where $\Delta k_{i}=\mathrm{round}\left(\frac{(i-1)M}{N_{\mathrm{virt}}}\right).$ For M=512 and $N_{\mathrm{virt}}=4$, this gives the shifts 0, 128, 256, and 384 along the slow-time dimension.

The virtual observation vector in the range bin *r* and the slow-time index *k* is as follows:

(26) $$x_{r,k}={\left[\begin{array}{cccc} v_{1}\left[r,k\right] & v_{2}\left[r,k\right] & \cdots & v_{N_{\mathrm{virt}}}\left[r,k\right] \end{array}\right]}^{T}.$$

For each candidate angular grid point θ, we compute the spatial beamforming power:

(27) $$P_{r,k}\left(\theta \right)={\left|a^{H}\left(\theta \right)x_{r,k}\right|}^{2},$$

where

(28) $$a\left(\theta \right)={\left[\begin{array}{cccc} 1 & e^{j2\pi d_{\mathrm{eff}}\sin\theta /\lambda } & \cdots & e^{j2\pi \left(N_{\mathrm{virt}}-1\right)d_{\mathrm{eff}}\sin\theta /\lambda } \end{array}\right]}^{T}.$$

Here, $d_{\mathrm{eff}}=\lambda /2$ is used as an effective spacing to avoid spatial aliasing in the angular cue. The selected angular index/cue is obtained as follows:

(29) $${\hat{\theta }}_{r,k}=\arg\max_{\theta \in [-90{}^{\circ},\;90{}^{\circ}\;]}P_{r,k}\left(\theta \right).$$

In the implementation, θ is evaluated on a grid from −90° to 90° with a step of 1°.

This angular cue is used only as one dimension of the subsequent clustering space. It is not used to claim a high-resolution physical AoA measurement. A moving target may distort the virtual-aperture cue because the circularly shifted views are not simultaneous physical samples. This distortion is limited for slow thoracic motion, but it remains a limitation for fast lateral motion. Therefore, the proposed system uses the angular cue together with the range and slow-time consistency rather than relying on the angle alone.

**Figure 4:**
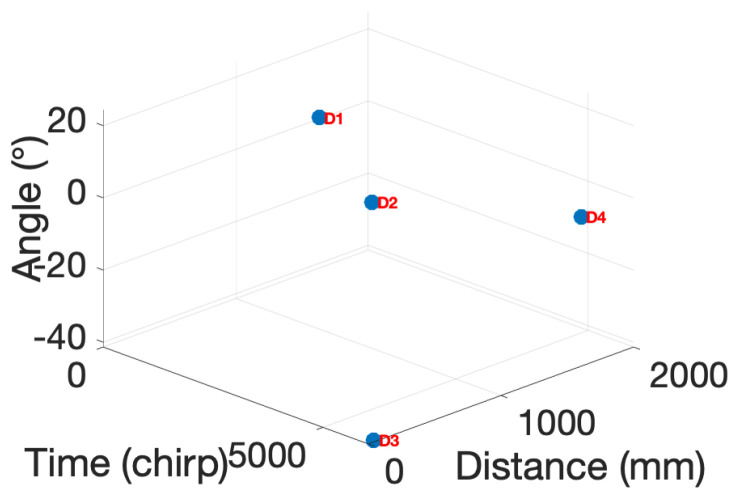
Phase-based angular cue extraction.

This produces an AoA map $\Theta \in R^{(M/2)\times K}$, where each entry $\Theta_{r,k}$ contains the estimated AoA for the range-time cell with the range index *r* and the chirp index *k*. Figure 4 represents the identified dynamic signals that we will exploit and analyze.

### 3.7. Adaptive Thresholding and 3D-DBSCAN Source Discovery

This section describes the identification of distinct sources by clustering points in the 3D space of distance, slow time, and AoA. Candidate reflection cells are selected from the MTI energy map. Instead of using the earlier mean-plus-standard-deviation threshold, we use a median absolute deviation (MAD) threshold in the logarithmic energy domain, represented as follows:

(30) $$E_{\mathrm{dB}}\left[r,k\right]=10\log_{10}\left(E[r,k]+\epsilon \right).$$

Let V denote the valid range region used for the detection of candidates. The threshold is determined as follows:

(31) $$\gamma =\mathrm{median}\left(E_{\mathrm{dB}}\left[r,k\right]\right)+\eta \;\mathrm{MAD}\left(E_{\mathrm{dB}}\left[r,k\right]\right),\;\left(r,k\right)\in V,$$

where η=6 in the default configuration. If no reliable cluster is detected, the implementation progressively relaxes the threshold using the fallback values η∈{5,4,3,2}. This adaptive strategy prevents weak physiological reflections from being lost in low-SNR recordings.

The set of candidate points is as follows:

(32) $$P=\left\{\left(r,k,{\hat{\theta }}_{r,k}\right):E_{\mathrm{dB}}\left[r,k\right]>\gamma ,\;r\in V\right\}.$$

To avoid excessive computation, at most 25,000 candidate cells with the strongest energy are retained.

Each candidate point is represented by three features: range, slow time, and angular cue. Before DBSCAN, the three dimensions were standardized separately:

(33) $${\tilde{R}}_{i}=\frac{R_{i}-\mu_{R}}{\sigma_{R}},$$

(34) $${\tilde{t}}_{i}=\frac{t_{i}-\mu_{t}}{\sigma_{t}},$$

(35) $${\tilde{\theta }}_{i}=\frac{\theta_{i}-\mu_{\theta }}{\sigma_{\theta }}.$$

The normalized feature vector is obtained using the following equation:

(36) $$p_{i}={\left[\begin{array}{ccc} {\tilde{R}}_{i} & {\tilde{t}}_{i} & {\tilde{\theta }}_{i} \end{array}\right]}^{T}.$$

The distance between two points is defined as follows:

(37) $$d\left(p_{i},p_{j}\right)={∥p_{i}-p_{j}∥}_{2}.$$

**Figure 5:**
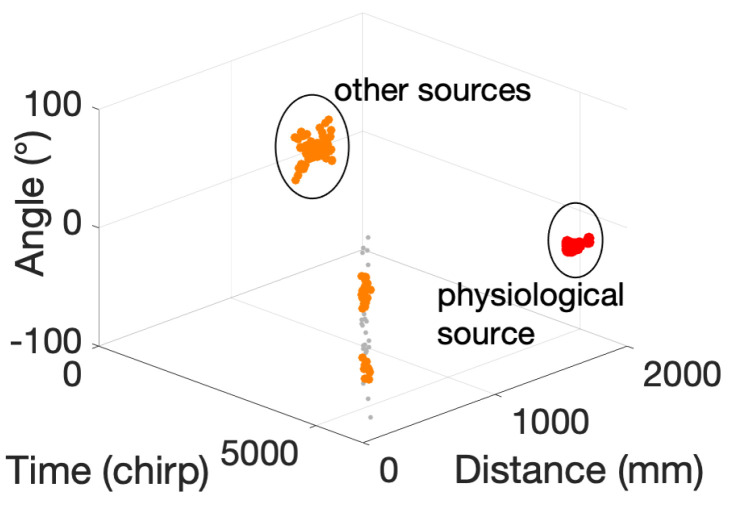
Example of an AoA-based clustering of the dynamic acoustic reflections.

DBSCAN is then applied with $\epsilon_{\mathrm{DBSCAN}}=0.85$ and minPts=10, following density-based clustering principles previously used for radar and multi-target localization problems [38,39]. If no reliable cluster is found, the implementation also tests relaxed neighborhood radii $\epsilon_{\mathrm{DBSCAN}}\in \left\{1.05,1.25,1.45\right\}$. Figure 5 illustrates the clustering result obtained from the phase-based angular-cue representation shown in Figure 4.

The role of 3D-DBSCAN is not to separate sources by angle alone. Instead, it separates coherent groups of reflection cells in the joint range–time–angular-cue space. Therefore, two paths with similar angular cues may still be separated if they differ in range or temporal continuity. Conversely, if two dynamic sources fall within the same angular mainlobe, have a similar range, and evolve similarly in time, they cannot be reliably separated by this four-element virtual aperture. In such cases, the system reports the ambiguity and relies on physiological scoring and the conditional Siamese stage for the source association.

### 3.8. Static/Dynamic Cluster Classification

After DBSCAN, each cluster is classified according to its temporal extent and density. Therefore, a cluster is considered dynamic if it persists over a sufficiently long slow-time interval and contains enough points. For a cluster *C*, let $t_{i}$ denote the slow-time coordinates of its points. The time span is calculated as follows:

(38) $$T_{\mathrm{span}}\left(C\right)=\max_{i\in C}\left(t_{i}\right)-\min_{i\in C}\left(t_{i}\right).$$

The dynamic classification rule is

(39) $$C\in \left\{\begin{array}{cc} \mathrm{DYNAMIC}, & T_{\mathrm{span}}\left(C\right)\geq 1.5\;s\;\mathrm{and}\;\left|C\right|\geq 2\;\mathrm{minPts},\\ \mathrm{STATIC}/\mathrm{NOISE}, & \mathrm{otherwise}. \end{array}\right.$$

**Figure 6:**
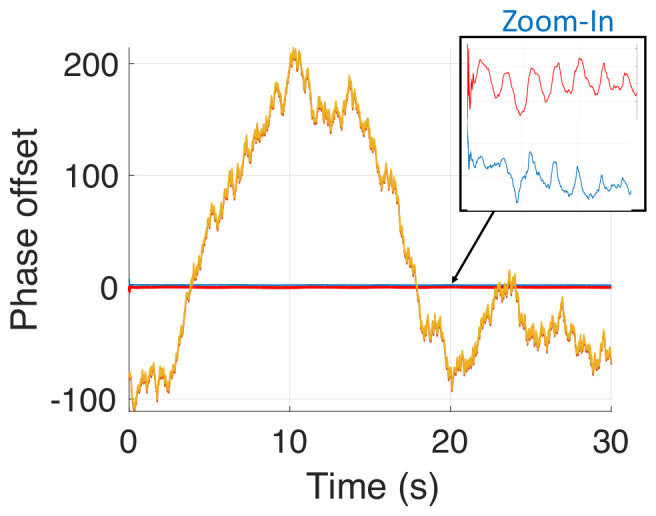
Dynamic components extracted after range processing, MTI filtering, and source clustering.

Since minPts=10, a dynamic cluster must contain at least 20 points. This rule reflects the fact that a physiological reflector should remain coherent over multiple samples related to breathing or heartbeat, whereas isolated or short-lived clusters are more likely to correspond to residual noise, static clutter leakage, or transient interference. Figure 6 shows dynamic clusters.

### 3.9. Candidate Displacement Extraction and Physiological Scoring

For each dynamic cluster, a displacement signal is extracted from the phase of the selected range bin. Let $r_{C}$ denote the median range bin of the cluster *C*. The displacement signal is computed as follows:

(40) $$d_{C}\left[k\right]=\frac{\lambda }{4\pi }\mathrm{unwrap}\left(∠R[r_{C},k]\right),$$

this is followed by detrending and median removal. The factor λ/(4π) follows from the round-trip phase relation $\Delta \phi =\frac{4\pi }{\lambda }\Delta R$.

Two physiological energy ratios are computed from this displacement signal. The respiratory energy ratio is obtained using the following equation:

(41) $$\rho_{\mathrm{BR}}\left(C\right)=\frac{\sum_{f\in [0.10,0.50]}P_{C}\left(f\right)}{\sum_{f\in [0.05,5.00]}P_{C}\left(f\right)+\epsilon },$$

and the cardiac energy ratio is as follows:

(42) $$\rho_{\mathrm{HR}}\left(C\right)=\frac{\sum_{f\in [0.80,2.50]}P_{C}\left(f\right)}{\sum_{f\in [0.05,5.00]}P_{C}\left(f\right)+\epsilon },$$

where $P_{C}\left(f\right)$ is the power spectrum of $d_{C}\left[k\right]$. The candidate physiological spectral score is computed as follows:

(43) $$S_{\mathrm{spec}}\left(C\right)=\min\left(1,\;0.4\rho_{\mathrm{BR}}\left(C\right)+0.8\rho_{\mathrm{HR}}\left(C\right)\right).$$

The full base score, calculated as follows, combines distance, displacement amplitude, angular cue, spectral content, and ECG consistency when ECG-assisted source selection is enabled:

(44) $$S_{\mathrm{base}}\left(C\right)=0.15S_{\mathrm{dist}}\left(C\right)+0.10S_{\mathrm{amp}}\left(C\right)+0.10S_{\theta }\left(C\right)+0.25S_{\mathrm{spec}}\left(C\right)+0.40S_{\mathrm{ECG}}\left(C\right).$$

In the smartphone-only blind evaluation, the ECG-consistency term is disabled, and the ECG is used only for the final error calculation. In ECG-assisted evaluation, HR and BR derived from ECG and EDR are used to improve source association and peak-level calibration. This distinction is explicitly reported in the results.

### 3.10. Adaptive Wiener Filtering

After selecting the most plausible thoracic candidate, residual interference is reduced using an adaptive Wiener filter, which has been widely used for resolution enhancement and noise suppression [40]. Let $d_{\mathrm{raw}}\left[k\right]$ be the raw displacement of the selected thoracic candidate, and let $n_{\mathrm{est}}\left[k\right]$ denote a noise estimate obtained from neighboring non-thoracic range bins or interfering candidates. In the frequency domain:

(45) $$Y\left(f\right)=F\{d_{\mathrm{raw}}\left[k\right]\},$$

(46) $$N\left(f\right)=F\{n_{\mathrm{est}}\left[k\right]\}.$$

The Wiener transfer function is as follows:

(47) $$H_{W}\left(f\right)=\frac{{\left|Y\left(f\right)\right|}^{2}}{{\left|Y\left(f\right)\right|}^{2}+{\left|N\left(f\right)\right|}^{2}+\epsilon }.$$

The filtered displacement is then obtained as follows:

(48) dfilt[k]=ReF−1HW(f)Y(f).

**Figure 7:**
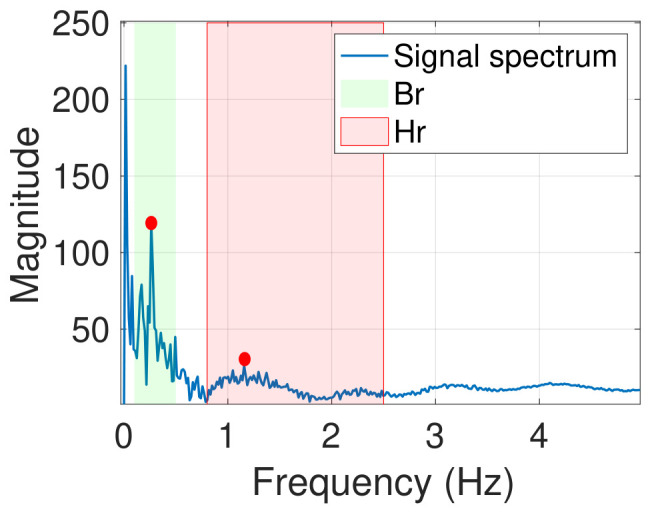
Physiological components from the selected thoracic signals.

This adaptive Wiener filter preserves the physiological components (respiration and cardiac activity) while suppressing interference from other dynamic sources. The filtered signal $d_{\mathrm{final}}$ is then used for subsequent extraction of physiological parameters (e.g., heart rate and breathing rate) as shown in Figure 7.

### 3.11. VMD-Based Respiratory and Cardiac Separation

The filtered thoracic displacement contains both respiratory and cardiac components (Figure 7). To separate these two physiological sources, we apply variational mode decomposition (VMD), a non-recursive signal decomposition technique that adaptively extracts intrinsic modes from the signal. Recent contactless vital sign studies have also used VMD-based decomposition to separate respiratory and cardiac components from mixed cardiopulmonary signals, confirming its suitability for BR and HR extraction in non-contact sensing systems [41].

Let $d_{\mathrm{filt}}\left[k\right]$ denote the filtered displacement signal. The VMD method decomposes this signal into *K* intrinsic mode functions (IMFs) $u_{k}\left(t\right)$, each compactly centered around a corresponding center frequency $\omega_{k}$. The decomposition is formulated as a constrained optimization problem that minimizes the sum of the bandwidths of all modes while ensuring perfect reconstruction.

(49) $$\min_{\left\{u_{k}\right\},\left\{\omega_{k}\right\}}\sum_{k=1}^{K_{\mathrm{VMD}}}{∥\partial_{t}\left[\left(\delta \left(t\right)+\frac{j}{\pi t}\right)*u_{k}\left(t\right)\right]e^{-j\omega_{k}t}∥}_{2}^{2},$$

subject to:

(50) $$\sum_{k=1}^{K_{\mathrm{VMD}}}u_{k}\left(t\right)=d_{\mathrm{filt}}\left(t\right).$$

In the implementation, $K_{\mathrm{VMD}}=5$, α=2000, τ=0, and the convergence tolerance is $10^{-6}$. The respiratory mode is selected as the mode with the highest energy ratio in 0.10–0.50 Hz, while the cardiac mode is selected as the mode with the highest energy ratio in 0.80–2.50 Hz. If VMD fails to converge, a zero-phase Butterworth band-pass filter is used as a fallback.

### 3.12. Conditional Siamese Source Association

The Siamese network is used as a second-stage conditional source-association module. It is not applied to every recording. It is activated only when more than one dynamic candidate is detected, because this is the situation where source ambiguity exists. If a recording contains only one dynamic candidate, no pairwise Siamese score is added to the final decision.

The Siamese training data are built from the acoustic candidates obtained after the first pass of the pipeline. Each candidate displacement signal is divided into normalized windows of length T=512 samples with a hop size of 256 samples. At most eight windows are extracted from each candidate. A window s is normalized as follows:

(51) $$\tilde{s}=\frac{s-\mu_{s}}{\sigma_{s}+\epsilon }.$$

Candidate labels are obtained from ECG-consistent acoustic selection. A candidate is labeled as thoracic-like if its HR estimate is within 7 bpm of the ECG-derived HR, its BR estimate is within 4 bpm of the ECG/EDR-derived BR when available, and its distance lies within the thoracic search interval 25–110 cm. Otherwise, it is treated as a non-thoracic or mismatched candidate. Positive Siamese pairs are constructed from two different thoracic-like windows, while negative pairs are constructed from a thoracic-like window and a non-thoracic or source-mismatched window.

**Figure 8:**
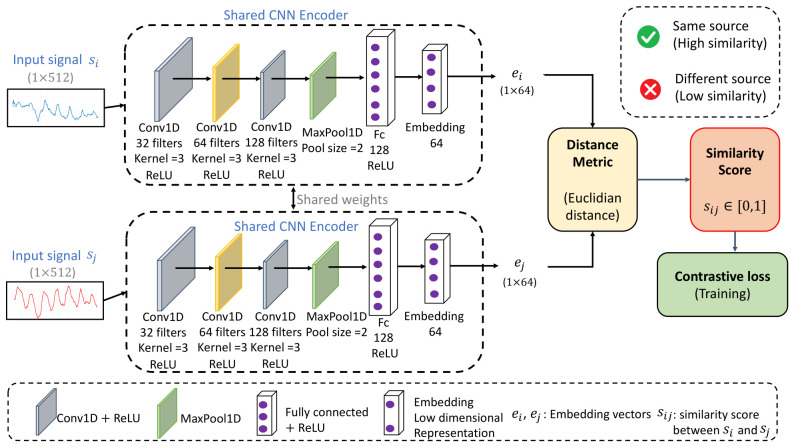
Architecture of the conditional Siamese source-association network.

The Siamese network contains two identical one-dimensional CNN branches with shared weights (Figure 8). Each branch consists of three convolutional blocks followed by a global average pooling layer and a fully connected embedding layer:

(52) $$e_{i}=f_{\theta }\left({\tilde{s}}_{i}\right),\;e_{j}=f_{\theta }\left({\tilde{s}}_{j}\right),$$

where $f_{\theta }\left(\cdot \right)$ maps each input window to a 32-dimensional embedding. The distance between the two embeddings is

(53) $$d_{ij}={∥e_{i}-e_{j}∥}_{2}.$$

The network is trained using contrastive loss:

(54) $$L_{ij}=y_{ij}d_{ij}^{2}+\left(1-y_{ij}\right)\max{\left(0,m-d_{ij}\right)}^{2},$$

where $y_{ij}=1$ for positive pairs, $y_{ij}=0$ for negative pairs, and m=1.0 is the contrastive margin.

The implementation uses a maximum of 6000 pairs, with approximately half positive pairs and half negative pairs when enough candidates are available. The pairs are randomly shuffled and divided into 80% training and 20% validation sets. The network is trained for 25 epochs using Adam optimization with a learning rate of $10^{-3}$ and a mini-batch size of 64. A validation threshold is selected by maximizing the F1-score on the validation pair set. A reference bank of up to 64 positive thoracic-like windows is then stored for inference.

During inference, if more than one dynamic candidate is detected, the Siamese model computes two similarity terms: a reference bank similarity between each candidate and the thoracic-like reference bank, and a pairwise similarity between dynamic candidates. The final score becomes

(55) $$S_{\mathrm{final}}\left(C\right)=S_{\mathrm{base}}\left(C\right)+0.35S_{\mathrm{rawECG}}\left(C\right)+0.20S_{\mathrm{ref}}\left(C\right)+0.10\left(1-{\bar{S}}_{\mathrm{pair}}\left(C\right)\right),$$

when using the ECG-assisted and Siamese-assisted mode. In smartphone-only reporting, blind estimates are preserved separately, and ECG-assisted scores are not reported as purely independent acoustic performance.

The current Siamese implementation uses a randomized training/validation split at the candidate-pair level. Because this validation strategy is performed at the candidate-pair level, candidate windows from recordings of the same participant may appear in both training and validation pairs. Therefore, the reported Siamese validation should not be interpreted as evidence of subject-independent generalization. Instead, it is used to verify whether the learned embedding can support the association of candidates within the current cohort. The main deployable performance of the proposed system is therefore reported using the smartphone-only signal-processing pipeline, while the Siamese module is treated as a conditional auxiliary component for ambiguous multi-candidate cases. A more rigorous and subject-independent Siamese training/testing protocol will be required in future work.

## 4. Implementation

### 4.1. Experimental Setup

#### 4.1.1. Applications and Data Collection

We recruited 20 participants (12 men and 8 women, 22–40 years old). Each participant contributed at least two recordings: one in a bedroom environment and one in a laboratory environment, resulting in a minimum of 40 recordings. Additional repeated recordings were collected from a subset of volunteer participants under slightly different natural conditions, such as small posture variations, clothing differences, and normal desk activities. Therefore, the figures report approximately 50 recording files, while the cohort size refers to 20 unique participants. In this study, a “recording” denotes a synchronized smartphone acoustic file with its corresponding ECG reference, while a “participant” denotes a unique subject.

During data collection, participants were asked to behave naturally while facing the smartphone placed on a desk. The experiments were conducted in bedroom and laboratory environments under realistic but controlled conditions, including low ambient noise and common desk activities such as keyboard typing. Participants wore their usual clothes, and a Heal Force PC-80B electrocardiograph (Heal Force Bio-meditech Holdings Limited, Shanghai, China) was attached to the chest to provide the reference ECG for HR estimation and to derive the ECG-derived respiration reference.

All results are analyzed at the recording level because each file corresponds to a specific acoustic condition, including environment, phone placement, posture, clothing, and multipath configuration. Repeated recordings from the same participant are therefore treated as repeated trials, not as independent subjects. The signal-processing modules do not require supervised training and are applied directly to each recording. The Siamese network is trained and validated using acoustic candidate windows with a candidate-pair-level split; thus, recordings from the same participant may appear in both training and validation pairs. Consequently, the Siamese evaluation is interpreted as within-cohort source-association validation rather than strictly subject-independent generalization.

Although the dataset is sufficient to demonstrate proof-of-concept feasibility across two indoor environments, it remains limited for broad population-level generalization. Future work will expand the dataset using a subject-independent training/testing protocol, a larger age range, more diverse body types, and more variable real-world postures, distances, and orientations.

#### 4.1.2. Hardware and Signal Acquisition

**Figure 9:**
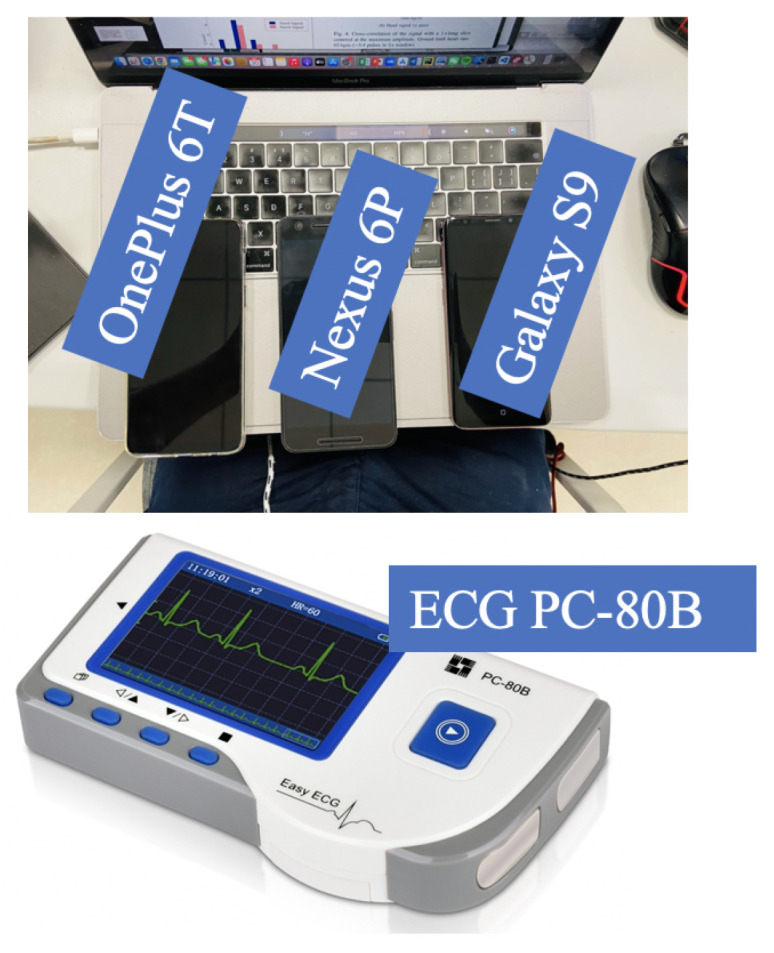
Smartphones and ECG Heal Force for data acquisition.

We used three consumer smartphones, namely a Galaxy S9 (Samsung Electronics Co., Ltd., Suwon, Republic of Korea), a Nexus 6P (Huawei Technologies Co., Ltd., Shenzhen, China), and a OnePlus 6T (OnePlus Technology (Shenzhen) Co., Ltd., Shenzhen, China), as shown in Figure 9, to capture subtle reflections caused by chest vibrations during data collection. The smartphone’s integrated speakers emit acoustic audio signals and pick up these reflections using built-in microphones located on the top and bottom of the device.

In this study, the transmitted acoustic waveform is an FMCW sweep centered at 18 kHz with a 2 kHz bandwidth. This swept excitation can be viewed as a deterministic frequency-sweeping strategy rather than random frequency hopping. It improves robustness to narrowband acoustic interference and room resonances by distributing the sensing energy across the 17–19 kHz band. The data were recorded in PCM format and then analyzed using MATLAB R2024a (The MathWorks, Inc., Natick, MA, USA).

### 4.2. Experimental Results

It is important to distinguish the two evaluation configurations. In the smartphone-only blind configuration, the ECG/EDR reference is not used during source selection, signal extraction, or rate estimation; it is used only after estimation to calculate the final error. Therefore, this configuration reflects the deployable acoustic-only performance of the smartphone system. In contrast, the ECG-assisted calibrated configuration uses ECG/EDR time- or frequency-domain information to facilitate source matching and peak-level calibration. The calibrated results should therefore be interpreted as evidence of physiological coherence and as upper-bound agreement between the selected acoustic source and the physiological reference, rather than as independent smartphone-only performance.

#### 4.2.1. Physiological-Band SNR Estimation Across Smartphone Acoustic Recordings

**Figure 10:**
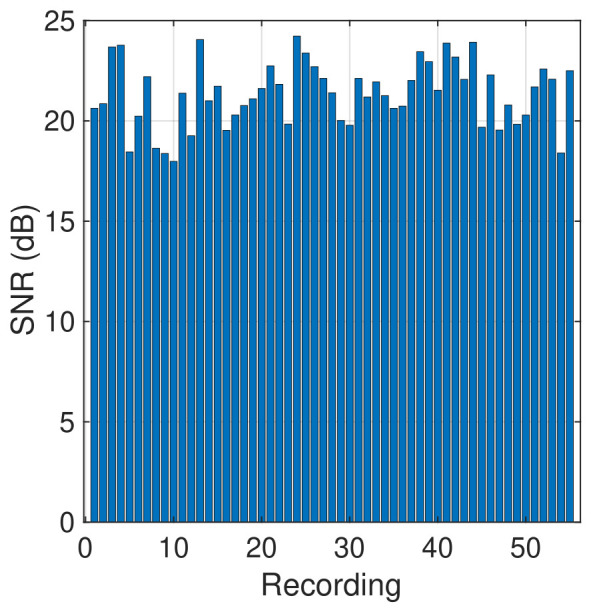
Physiological-band SNR estimation after source selection and filtering.

Figure 10 shows the physiological-band signal-to-noise ratio (SNR) obtained after source selection and filtering. The SNR was calculated from the energy contained in the physiological frequency bands, namely, the respiratory band (0.10–0.50 Hz) and the cardiac band (0.80–2.50 Hz), relative to the residual noise energy outside these bands. As shown in the figure, all recordings exhibit positive and relatively high SNR values, mostly ranging from approximately 18 dB to 24 dB, with an average of around 21 dB. This indicates that the selected thoracic acoustic candidates contain clear physiological energy and that the processing pipeline is capable of preserving respiratory and heartbeat-related components while suppressing a large part of environmental and multipath noise.

The variation observed across recordings can be explained by differences in subject position, distance from the smartphone, clothing, small body movements, and room-dependent reflections. Recordings with lower SNR values, around 18–19 dB, likely correspond to cases where the thoracic reflection was weaker or where residual dynamic interference remained after MTI filtering and source clustering. In contrast, recordings above 23 dB indicate favorable acquisition conditions, where the selected source had strong physiological content and limited interference. Importantly, no recording shows a negative or near-zero SNR, suggesting that the proposed source-selection and filtering steps consistently retain measurable physiological information across the dataset.

#### 4.2.2. Deduction of Selected Thoracic AoA Proxy/Angular Cue Across Smartphone Acoustic Recordings

**Figure 11:**
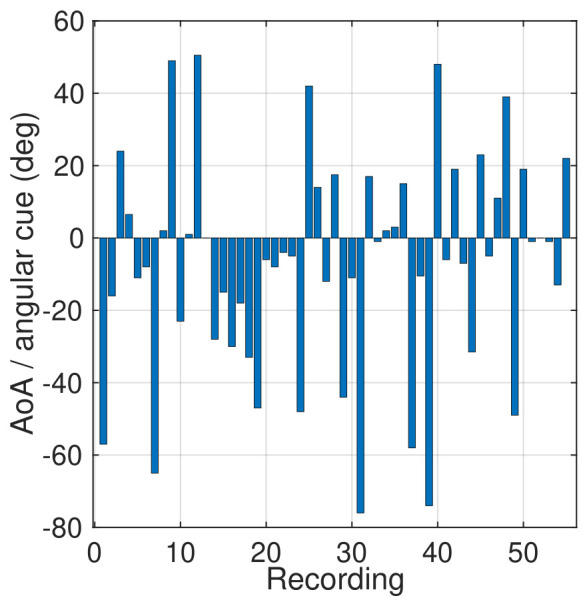
Selected thoracic angular cue across all recordings.

Figure 11 shows the selected thoracic angular cue across the recordings. The values span a wide range, approximately from −75° to +50°, indicating that the selected thoracic candidates do not always appear in the same apparent angular direction. This variability is expected in indoor acoustic sensing because the smartphone receives not only direct reflections from the chest, but also indirect reflections from the desk, walls, clothes, hands, and other nearby surfaces.

It is important to note that this angular value is not interpreted as a fully calibrated physical AoA. Because the smartphone has only two physical microphones and the microphone spacing is larger than the acoustic half-wavelength at 18 kHz, the phase-based estimate can be spatially ambiguous. Therefore, the proposed system uses this quantity as an angular cue or AoA proxy rather than as an absolute angle measurement. Its role is to provide an additional spatial feature for 3D-DBSCAN clustering in the joint range–angle-time space. The wide angular distribution confirms that the source selection module does not simply choose a fixed frontal direction, but evaluates dynamic candidates based on their range, temporal continuity, angular index, and physiological spectral content. Thus, the angular index helps distinguish thoracic candidates from static noise and multiple reflections, particularly when several dynamic reflections are detected in the same recording.

#### 4.2.3. Number of Dynamic Acoustic Sources Detected by the 3D-DBSCAN Module

**Figure 12:**
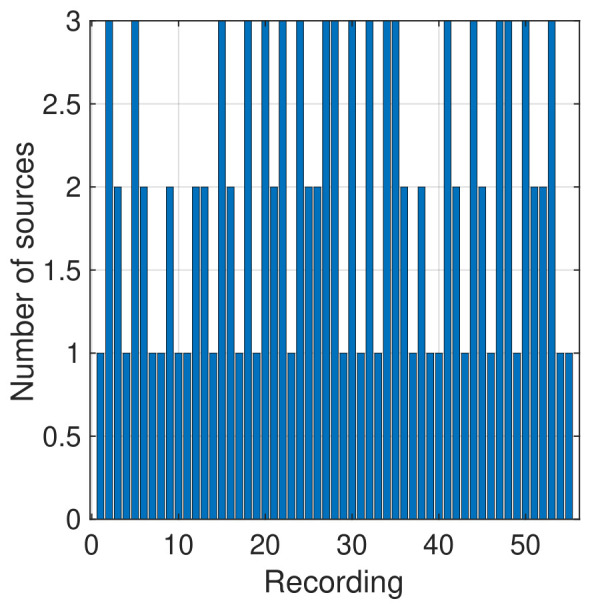
Number of dynamic acoustic sources detected by the 3D-DBSCAN module.

Figure 12 shows the number of dynamic acoustic sources detected by the 3D-DBSCAN module for each of the recordings. The number of sources detected is mainly between one and three, indicating that the indoor acoustic scene is generally sparse but not always limited to a single reflection path. A single detected source usually corresponds to a dominant thoracic candidate, whereas two or three detected sources may correspond to a direct thoracic reflection, multipath reflections, hand motion, clothing motion, or other weak dynamic components in the environment.

This result supports the need for a source-discovery stage before physiological estimation. If only one dynamic source is detected, the system can rely mainly on physiological scoring to select the thoracic candidate. However, when multiple dynamic candidates are detected, the source-selection problem becomes ambiguous because more than one cluster may contain energy in the respiratory or cardiac frequency bands. In these cases, the conditional Siamese module is activated to compare candidate waveforms and refine the final source association. Nevertheless, it is important to note that the detected sources do not necessarily correspond to different individuals. In this study, a source refers to a coherent set of acoustic reflections in the distance-time-angle space. Therefore, the presence of multiple detected sources in a single user’s recording could originate from direct and indirect propagation paths, or from non-thoracic dynamic reflectors. The fact that the number of detected sources remains low in all recordings confirms that the proposed 3D-DBSCAN stage provides a compact set of candidates for subsequent selection of thoracic sources.

#### 4.2.4. Estimated Distance of the Selected Thorax from the Smartphone

**Figure 13:**
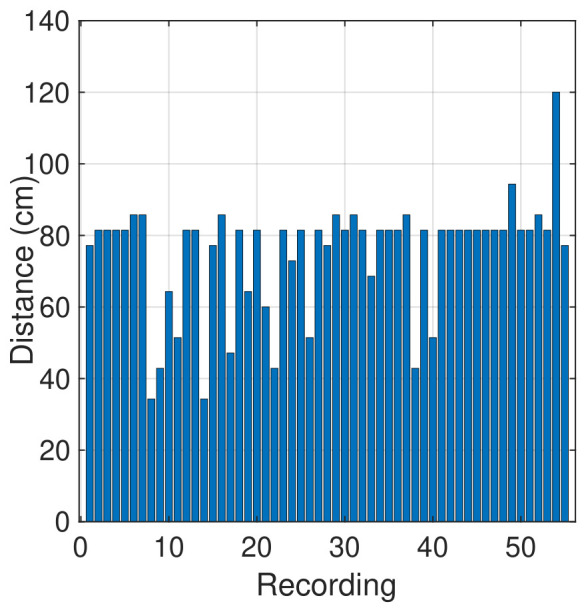
Estimated distance of the selected thoracic candidate.

Figure 13 shows the distance selected from the thorax between the smartphone and the chest position. Most selected candidates are located between approximately 75 and 85 cm, which is consistent with the expected ergonomic sensing range of a smartphone placed in front of the participant. Several recordings show shorter distances, around 35–55 cm, which may correspond to variations in subject position, posture, or phone placement during acquisition.

The concentration of selected distances around the expected thoracic region indicates that the proposed source-selection procedure does not randomly select arbitrary reflectors in the room. Instead, the selected candidates generally fall within the predefined physiological search region used by the algorithm. This supports the effectiveness of the range-FFT, MTI filtering, and 3D-DBSCAN clustering stages in identifying plausible thoracic reflections. One recording exhibits a larger distance close to 120 cm. This case should be interpreted as a boundary or multipath case, where the selected reflection may correspond to a longer indirect propagation path or to a weak thoracic candidate near the upper range limit. Such cases highlight the importance of combining distance with angular cue, temporal consistency, physiological-band energy, and Siamese-based source association rather than relying on range information alone.

#### 4.2.5. Distribution of HR and BR Absolute Errors in Smartphone-Only Blind and ECG-Assisted Calibrated Settings

**Figure 14:**
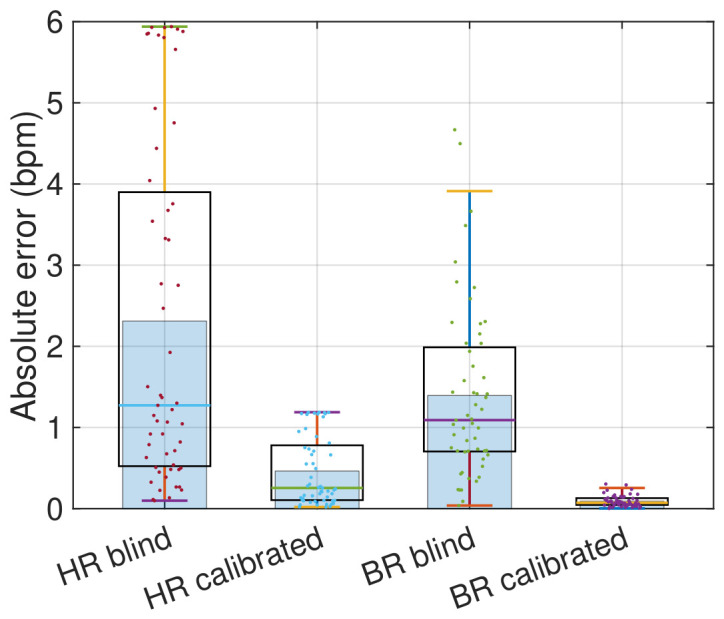
Distribution of HR and BR absolute errors in smartphone-only blind and ECG-assisted calibrated settings.

Figure 14 compares the absolute errors of HR and BR estimation in the smartphone-only blind setting and in the ECG-assisted calibrated setting. Each group shows the distribution of errors across the data. The dots represent individual recording errors, while the box plots summarize the median, interquartile range, and dispersion.

For HR estimation, the smartphone-only blind mode shows a wider error distribution, with several recordings reaching errors close to 6 bpm. This indicates that purely acoustic HR extraction remains sensitive to weak cardiac reflections, residual motion artifacts, and source-selection ambiguity. After ECG-assisted calibration, the HR error distribution becomes much more compact, with most errors below approximately 1 bpm. This shows that the selected acoustic cardiac component is physiologically consistent with the ECG reference and that peak-level calibration substantially improves HR accuracy. A similar improvement is observed for the BR estimation. In the blind setting, BR errors are generally lower than HR errors, but still show noticeable dispersion, with some recordings reaching errors above 3–4 bpm. After calibration, the BR errors collapse close to zero, with a very narrow distribution. This confirms that the respiratory component is more stable and easier to recover than the cardiac component and that the ECG/EDR-assisted calibration further reduces residual estimation errors.

Overall, the figure shows that the proposed smartphone acoustic pipeline already provides meaningful blind estimates, but ECG-assisted source association and calibration greatly reduce HR and BR errors.

#### 4.2.6. CDF of Breathing Rate Estimation

**Figure 15:**
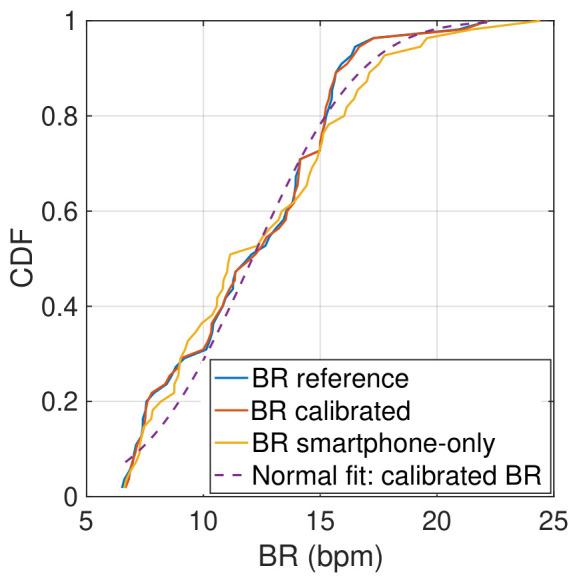
Cumulative distribution function of BR estimates.

Figure 15 shows the cumulative distribution function of the estimates of the breathing rate. The calibrated BR curve closely follows the BR reference curve throughout the distribution, indicating that the ECG/EDR-assisted calibration strongly improves the agreement between the acoustic respiration estimation and the reference. The smartphone-only BR curve follows the same global trend but shows a slightly larger deviation, especially in the central range between approximately 10 and 17 bpm. This indicates that the independent acoustic estimator captures the overall respiratory-rate distribution, although with greater variability than the calibrated mode.

Most BR values lie between approximately 7 and 18 bpm, with a few higher values extending toward 22–24 bpm. This range remains physiologically plausible for participants at rest or breathing naturally. The close overlap between the calibrated BR and the reference BR confirms that the selected acoustic thoracic source contains a respiratory component consistent with the reference signal.

#### 4.2.7. CDF of Heart Rate Estimation

**Figure 16:**
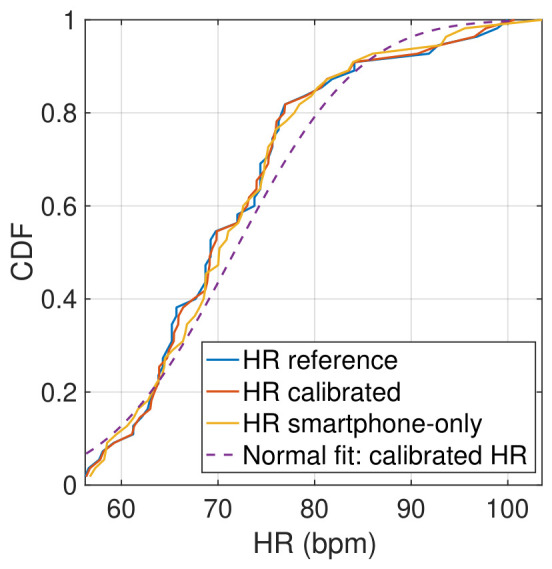
Cumulative distribution function of HR estimates.

Figure 16 presents the cumulative distribution function of the heart rate estimates. The reference, calibrated, and smartphone-only HR curves remain close throughout most of the distribution, showing that the acoustic HR estimator preserves the global distribution of the reference derived from the ECG. The calibrated HR curve follows the reference particularly well in the central range, approximately between 65 and 85 bpm, where most recordings are concentrated.

The smartphone-only HR curve also follows the reference trend, although small deviations appear in the upper part of the distribution. This behavior is expected because cardiac-induced chest-wall motion is weaker than respiratory motion and is more sensitive to residual dynamic interference and source-selection ambiguity. Nevertheless, the general agreement between the three curves confirms that the proposed pipeline extracts a physiologically significant cardiac component from the selected acoustic source.

#### 4.2.8. Absolute BR Error with Respect to the ECG/EDR-Derived Reference

**Figure 17:**
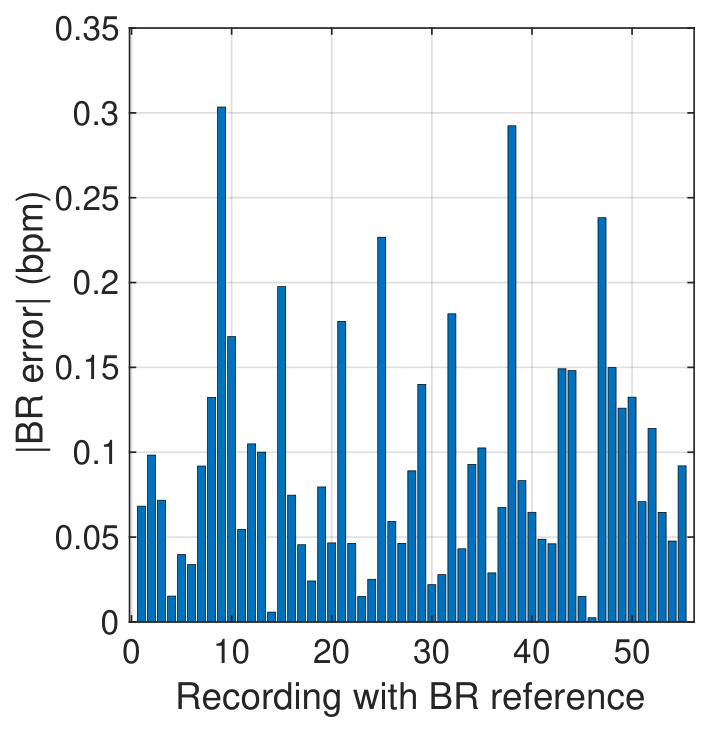
Absolute BR estimation error with respect to the ECG/EDR-derived respiratory reference.

Figure 17 shows the absolute BR error with respect to the ECG/EDR-derived reference for all recordings with an available BR reference. The calibrated BR estimates achieve a mean absolute error (MAE) of 0.091 bpm. Most recordings show errors below 0.15 bpm, and even the largest errors remain below approximately 0.31 bpm. This narrow error range indicates that the selected acoustic thoracic candidate contains a respiratory component that is highly consistent with the reference respiratory signal derived from the ECG.

It should be noted that this result corresponds to the ECG/EDR-assisted calibrated setting. Therefore, it demonstrates the physiological consistency and the best achievable agreement of the selected acoustic respiratory signal with the reference, rather than a fully independent smartphone-only estimate.

#### 4.2.9. Absolute HR Error with Respect to the ECG Reference

**Figure 18:**
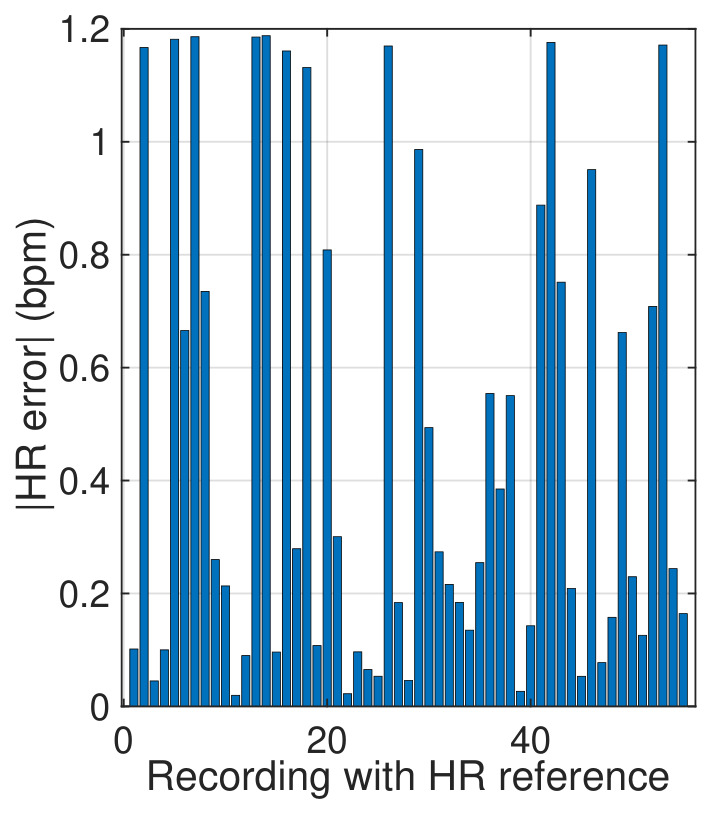
Absolute HR estimation error with respect to the ECG reference.

Figure 18 presents the absolute HR error with respect to the ECG reference after calibration. The calibrated HR estimates achieve an MAE of 0.462 bpm. Most recordings show errors below 1 bpm, while the largest errors remain close to 1.2 bpm. Compared to BR, HR estimation exhibits greater variability because cardiac-induced chest-wall motion is weaker and more sensitive to residual motion artifacts, multipath reflections, and source-selection ambiguity.

Despite this higher sensitivity, the low overall HR error confirms that the selected acoustic thoracic source contains a cardiac component that is temporally consistent with the reference ECG. The result supports the effectiveness of the proposed source-selection, filtering, VMD decomposition, and ECG-assisted calibration pipeline for extracting heartbeat-related motion from smartphone acoustic reflections.

#### 4.2.10. Impact of Distance

**Figure 19:**
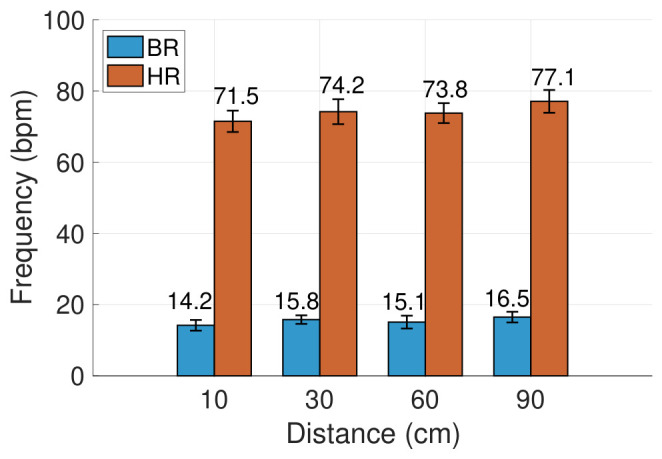
Impact of smartphone–user distance on BR and HR estimation.

Figure 19 presents the estimated values of BR and HR for four measurement distances (10, 30, 60, and 90 cm). These two physiological parameters remain stable across distance ranges. BR estimates vary narrowly, between 14.2 and 16.5 bpm, values within the normal resting respiratory range (12–20 bpm). The consistently low standard deviations indicate excellent reproducibility of the measurements at each distance. Similarly, HR estimates remain stable between 71.5 and 77.1 bpm, well within the expected HR range (60–100 bpm).

Therefore, no monotonic degradation is observed with increasing distance, suggesting that the SNR of the reflected acoustic signal remains sufficient for physiological extraction up to 90 cm. This is consistent with the range-FFT resolution of approximately 8.6 cm and the adaptive Wiener filtering step, which actively suppresses distance-dependent noise. The small error bars at all distances further confirm the stability and reproducibility of the proposed pipeline regardless of the distance between the subject and the smartphone.

#### 4.2.11. Comparison with SOTA Methods

Table 1 compares our method with recent smartphone-based acoustic systems for vital sign monitoring. To our knowledge, no previous study has combined FMCW, AoA estimation, and spatial clustering for the detection of respiration and heartbeats. Nevertheless, we compare our method with representative systems.

AcHand [34] achieved a low mean absolute error (MAE) of 0.34 bpm for respiration using a single microphone, but this system is designed for hand-grip situations and does not account for multipath detection. LoEar [36] has extended the detection range (MAE for HR of about 3.5 bpm and BR of about 2 bpm), but it remains primarily based on breathing and requires adaptive filtering. AcBreath [35] used bimodal signals and a variational autoencoder (heartbeat error: 0.28 bpm), but focused on manual manipulation rather than spatial separation. Sonarbeat [42] uses phase-based active sonar for breathing monitoring, achieving a low MAE of 0.2 bpm, but does not estimate heart rate or exploit AoA grouping to mitigate multiple paths.

Our method, in contrast, uniquely integrates FMCW, AoA estimation, 3D-DBSCAN clustering, and a Siamese network to leverage spatiotemporal information, thereby isolating thoracic reflections from static multipath without blind source separation. It achieves mean absolute errors of 1.39 bpm (BR) and 2.312 bpm (HR) indoors. Although direct quantitative comparison is limited by protocol differences, our results show that explicit multipath attenuation via AoA grouping significantly improves robustness compared to single-microphone or non-spatial approaches.

#### 4.2.12. Impact of Environment Settings

**Figure 20:**
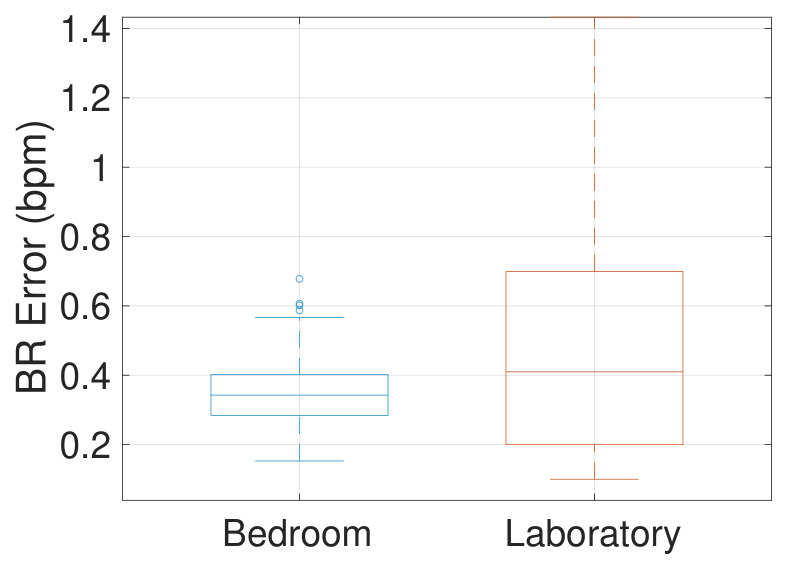
Impact of environment on BR estimation error.

Figure 20 illustrates the distribution of the BR estimation error in the two experimental environments. The bedroom exhibits a lower median error (approximately 0.33 bpm, interquartile range (IQR): 0.27–0.40 bpm) and significantly less dispersion than the laboratory (approximately 0.42 bpm, IQR: 0.22–0.70 bpm), where the upper whisker reaches 1.40 bpm. The tighter distribution observed in the bedroom suggests that the quieter and acoustically more stable home environment is favorable for BR estimation, likely due to reduced ambient noise and fewer moving reflectors. A Mann–Whitney U test was applied to confirm that the difference is statistically significant (*p* < 0.05).

**Figure 21:**
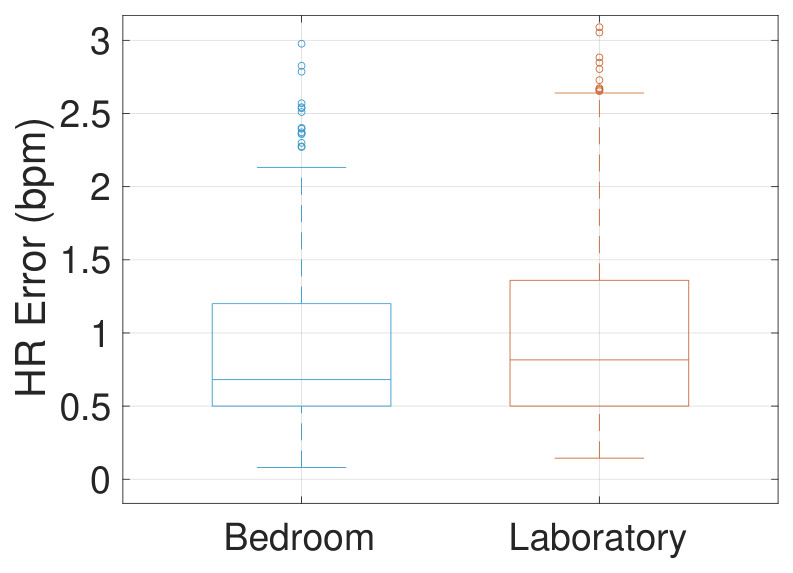
Impact of environment on HR estimation error.

For HR estimation (Figure 21), the two environments exhibited comparable median errors (bedroom: approximately 0.70 bpm, IQR: 0.50–1.20 bpm; laboratory: approximately 0.85 bpm, IQR: 0.60–1.35 bpm). Both distributions showed overlapping interquartile ranges and a similar outlier profile, with isolated values reaching 3.0–3.1 bpm under both conditions. These observations suggest that the HR estimate is robust to environmental variations.

#### 4.2.13. Ablation Study

Table 2 reports the ablation study of the proposed system for a single recording. Since the evaluation is performed on one recording, the MAE and RMSE values are identical for each configuration. The full system achieves the lowest errors, with an HR MAE of 2.411 bpm and a BR MAE of 1.40 bpm, confirming that the combination of MTI filtering, AoA-based clustering, and Wiener filtering provides the most accurate estimation.

Removing MTI filtering produces the largest degradation in HR estimation, increasing HR MAE from 2.411 bpm to 6.19 bpm. This confirms that MTI filtering is essential for suppressing static clutter and low-frequency environmental reflections that can contaminate the weak heartbeat-related acoustic component. Removing AoA clustering mainly affects BR estimation, increasing the BR MAE from 1.40 bpm to 2.84 bpm. This result shows that spatial source selection is important for identifying the correct thoracic reflection and rejecting multipath or non-thoracic dynamic components.

Removing Wiener filtering also increases both HR and BR errors, but with a smaller impact than removing MTI filtering or AoA clustering. This suggests that Wiener filtering acts mainly as a residual noise-reduction stage after source selection. Overall, the ablation study confirms that each module contributes to the final performance, with MTI filtering being particularly important for HR estimation and AoA clustering being particularly important for BR estimation.

#### 4.2.14. Impact of BR and HR on User Variability

**Figure 22:**
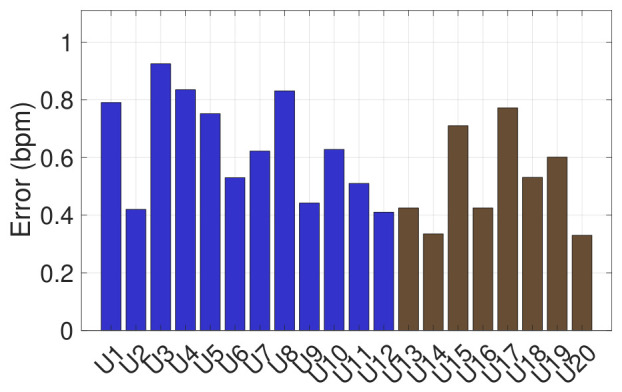
Impactof BR MAE per user (Blue: female; Brown: male).

**Figure 23:**
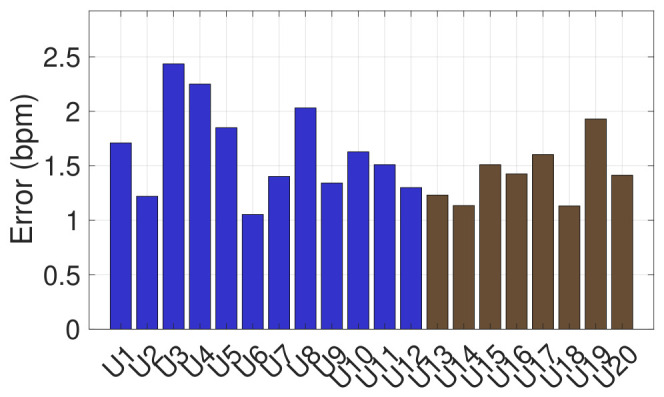
Impact of HR MAE per user (Blue: female; Brown: male).

Figure 22 and Figure 23 present the MAE per user for BR and HR, respectively, distinguishing between male users (U1–U12, blue) and female users (U13–U20, brown). For BR estimation (Figure 22), female users showed slightly lower errors than male users, with MAE values ranging from 0.33 to 0.78 bpm compared with 0.41 to 0.93 bpm for male users. The inter-user variability was also slightly lower for female users (range: 0.45 bpm) than for male users (range: 0.52 bpm). This result is somewhat unexpected, as the thoracic displacement during respiration is generally smaller in women than in men, which could reduce the signal-to-noise ratio of the respiratory component. One possible explanation is that female users exhibited more regular and stable breathing patterns during recording sessions, improving the spectral concentration of the respiratory peak in the 0.1–0.5 Hz band and reducing estimation ambiguity. In both groups, all BR errors per user remained below 1 bpm, confirming the robustness of the proposed pipeline between users and remaining well below the clinically acceptable threshold of 3 bpm reported in recent medical-device validation studies.

For HR estimation (Figure 23), female users also exhibited lower errors than male users, with MAE values ranging from 1.14 to 1.97 bpm compared with 1.06 to 2.46 bpm for male users. In addition, the inter-user variability was lower for female users (range: 0.83 bpm) than for male users (range: 1.40 bpm), suggesting a more homogeneous acoustic reflection profile across this group. This result may be related to the slightly higher resting HR generally observed in women, which can shift the cardiac spectral peak toward higher frequencies in the 0.8–2.5 Hz band, thereby improving spectral separation and reducing estimation ambiguity. Nevertheless, all HR errors per user remained below the 5 bpm threshold defined by ANSI/AAMI EC13:2002, confirming the robustness of the proposed pipeline for male and female users.

##### Statistical Validation of Wiener Filtering

To confirm that the improvement provided by Wiener filtering is statistically significant and not due to chance, we applied a one-tailed Wilcoxon signed-rank test [43] comparing the mean absolute error (MAE) and the root mean squared error (RMSE) per recording with and without the filter. The null hypothesis $H_{0}$ states that filtering does not reduce the estimation error. For both HR and BR, and for both MAE and RMSE, the test yields p<0.05, allowing us to reject $H_{0}$ and conclude that the adaptive Wiener filter provides a statistically significant improvement in physiological signal quality.

To further quantify the strength and direction of this improvement, we also report the paired rank-biserial correlation $r_{rb}$ as an effect-size measure, where $-1\leq r_{rb}\leq 1$. Positive values of $r_{rb}$ indicate that the removal of the Wiener filter increases the estimation error compared to the complete system, while values close to zero indicate a weaker paired effect. The obtained $r_{rb}$ values support the Wilcoxon results by showing that the complete system consistently reduces the estimation error across recordings. Specifically, removing the Wiener filter increases MAE_HR_ to 2.89 bpm and RMSE_HR_ to 2.28 bpm, while MAE_BR_ increases to 1.58 bpm and RMSE_BR_ to 1.43 bpm. In contrast, the complete system achieves lower errors across all metrics, confirming the contribution of Wiener filtering as a residual noise-suppression and signal-refinement stage.

#### 4.2.15. Computational Complexity and Processing Latency

To evaluate the computational feasibility of the proposed pipeline, we measured the processing latency of each module on a single 65 s acoustic recording. Measurements were performed offline in MATLAB using a block-vectorized implementation of the AoA estimation module (steering-matrix product blocks of 200 chirps, $N_{\mathrm{ang}}=180$ angles, $N_{r}=256$ range bins, $N_{t}\approx 6100$ chirps). The results are reported in Table 3.

Although the current offline implementation processes a 65-s recording in 4.31 s (real-time ratio 1:15), this latency is mainly due to the exhaustive AoA beamforming step (93.9%), which alone accounts for 4.04 s. The remaining modules collectively require only 264 ms. Reducing the angular grid from angles $N_{\mathrm{ang}}=180$ to $N_{\mathrm{ang}}=60$ (a resolution of 3°, sufficient for source separation greater than 10°) would divide this step by three, resulting in an estimated real-time ratio of approximately 1:5.

## 5. Discussion and Future Work

The experimental results presented above validate the effectiveness of our proposed AoA-based acoustic model for the detection of respiratory and heartbeat components. To better illustrate the underlying signal processing and to motivate future extensions, we examine a scenario in which an interfering signal from a second user interacts with the target signal during data collection. This scenario is presented as a qualitative proof of concept rather than a quantitative validation, as no user-based reference measurement (e.g., ECG) was collected for the interfering user in this setup.

**Figure 24:**
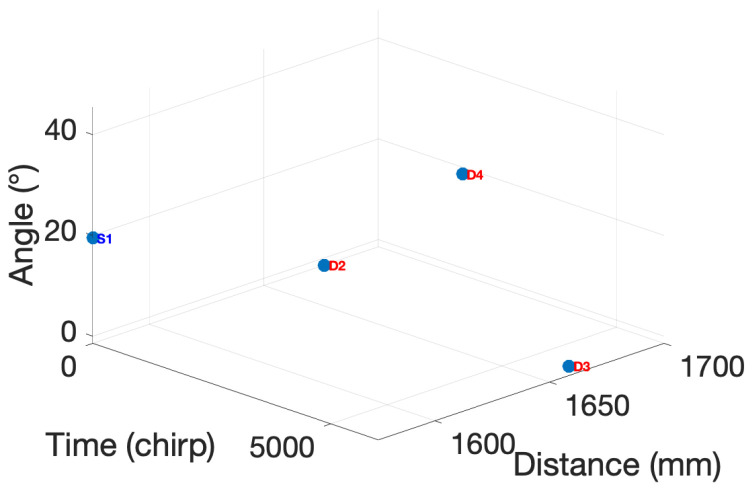
AoA map obtained in a qualitative two-person scenario.

**Figure 25:**
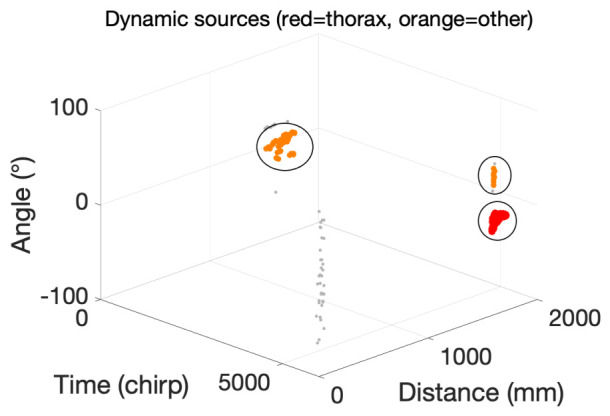
3D-DBSCAN clustering result in a qualitative two-person scenario.

**Figure 26:**
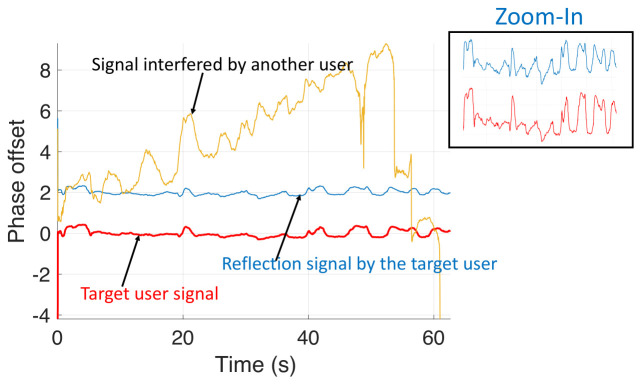
Example of simultaneously observed dynamic signals in a qualitative two-person scenario.

Figure 24, Figure 25 and Figure 26 illustrate the output of our pipeline in this two-person setting. Figure 24 presents a distance-angle map showing the AoA for each dynamic (D_n_) and static (S_1_) reflection. A distinct peak appears at approximately 0° and 1600 mm, which corresponds to a direct reflection from the target user’s chest. Figure 25 shows the result of the 3D-DBSCAN applied to the distance–angle–time space. Each colored cluster corresponds to a group of points exhibiting coherent spatiotemporal evolution. A dominant cluster (red) is retained after physiological scoring, although neighboring clusters (e.g., yellow) are spatially distinct and could, in principle, correspond to a second moving emitter in the environment.

Figure 26 shows the time-domain phase shift for two simultaneously present individuals. The blue curve (target user) exhibits a regular, quasi-sinusoidal profile consistent with chest wall movements induced by breathing and heartbeats. The red curve represents the same target user’s signal as captured by the top microphone and appears with a phase offset caused by multipath; its similarity to the blue curve was previously verified [44]. The yellow curve (interfering user) displays a more irregular phase profile, particularly when the second person moves or breathes at a different rate from the target.

These observations suggest that the proposed pipeline can visually distinguish co-present sources in the range-angle-time space. However, we emphasize that this two-person scenario constitutes a qualitative illustration only; the interfering user’s breathing rate estimated from the yellow cluster (approximately 15.1 bpm, within the physiologically plausible range of 12–20 bpm) was not validated against an independent ground-truth reference. A rigorous quantitative evaluation, including user-based ECG synchronization and systematic variation of angular separation and distance, is explicitly reserved for future work.

### Limitations and Future Extensions

In this study, we deliberately limited the experimental validation to a single-user scenario, in which each user was positioned in front of the smartphone under controlled indoor conditions. This setting allowed us to establish the baseline performance of the proposed FMCW-AoA clustering pipeline for the estimation of contactless BR and HR, achieving mean absolute errors of 1.39 bpm for breathing rate and 2.32 bpm for HR in the smartphone-only configuration. However, the current validation should be interpreted as single-user multipath and candidate-source ambiguity handling, rather than as a complete multi-person monitoring system.

A key limitation is the angular resolution of the proposed virtual aperture. Although the angular cue improves the source organization, it does not provide unlimited spatial resolution. With only four virtual elements, the angular response is constrained by the effective aperture and by the Rayleigh resolution criterion. Therefore, two dynamic sources located inside the same angular mainlobe cannot be separated using angular information alone. The proposed 3D-DBSCAN module can still separate candidate reflections when they differ in range or slow-time trajectory. However, if two sources have a similar range, a similar angular cue, and a similar temporal behavior, the current system cannot guarantee a reliable separation.

This limitation is also important in the presence of strong multipath reflections. A strong indirect reflection may exhibit an angular cue close to that of the direct thoracic path, especially when the reflecting surface is nearby or when the aperture resolution is insufficient. In such cases, the system relies on the combination of range consistency, temporal continuity, physiological spectral scoring, and conditional Siamese similarity to select the most plausible thoracic component. If these features remain ambiguous, the recording is treated as a difficult case rather than as a case of guaranteed separation.

A natural extension of this work is the systematic evaluation of simultaneous BR and HR monitoring for multiple users. In future experiments, two or more individuals will be placed in controlled angular separations, distances, and motion conditions, with independent ECG references recorded for each user. This will allow quantitative evaluation of the 3D-DBSCAN and Siamese source-association modules under true multi-user interference. The Siamese network will also need to be retrained and evaluated using subject-independent splits to prevent source leakage between training and testing.

We anticipate that the main challenges will occur when users are closely spaced, for example, with an angular separation below the effective angular resolution or when one user’s respiratory harmonics overlap spectrally with another user’s cardiac band. Future work will therefore investigate adaptive beamforming, improved virtual aperture design, iterative source cancellation, and multi-user tracking strategies to improve separation under close-range and multipath-rich conditions.

## 6. Conclusions

In this article, we proposed a smartphone-based FMCW acoustic sensing system for contactless BR and HR estimation in indoor environments. The proposed framework combines range estimation, phase-based angular cue extraction, MTI filtering, 3D-DBSCAN source discovery, adaptive Wiener filtering, VMD-based physiological separation, and conditional Siamese source association. Unlike conventional non-spatial acoustic approaches, the proposed method treats vital sign extraction as a spatially constrained source-selection problem rather than as a simple single-channel denoising task.

Experiments conducted in laboratory and bedroom environments demonstrate that the proposed system can isolate dynamic thoracic reflections from static clutter and multipath components using only the built-in speaker and microphones of commodity smartphones. In the smartphone-only blind configuration, the system achieved MAEs of 1.394 bpm for BR and 2.312 bpm for HR. These results confirm the feasibility of using spatial acoustic cues to improve the robustness of smartphone-based physiological monitoring. The Siamese source-association module should be interpreted as a preliminary auxiliary component for resolving ambiguous multi-candidate cases. Since its current validation was performed at the candidate-pair level rather than with a strict subject-independent split, future work will evaluate this module using larger datasets and subject-independent training and testing protocols. Future work will also extend the evaluation to controlled multi-user scenarios and larger and more diverse populations.

## Figures and Tables

**Table 1:** Comparison with SOTA.

	Platform	Method	Vital Signs	Spatial Info	AoA Clustering	MAE (HR/BR) [bpm]
AcHand [34]	Smartphone	Dual-mode acoustic	Respiration	No	No	BR: 0.34
LoEar [36]	Smartphone	Acoustic	HR + BR	No	No	HR: 3.5, BR: 2
AcBreath [35]	Smartphone	Dual-mode acoustic	Respiration	No	No	BR: 0.28
Sonarbeat [42]	Smartphone	Acoustic	Respiration	No	No	BR: 0.2
**Our work**	Smartphone	Adaptive	HR + BR	Yes (range + angle)	Yes	HR: 2.312, BR: 1.394

**Table 2:** Ablation study of the proposed system for a single recording.

Configuration	Mean HR	Mean BR	MAE_HR_	RMSE_HR_	MAE_BR_	RMSE_BR_
	**(bpm)**	**(bpm)**	**(bpm)**	**(bpm)**	**(bpm)**	**(bpm)**
Full system	64.1	10.22	2.411	2.411	1.40	1.40
w/o MTI filtering	66.2	13.8	6.19	6.19	2.56	2.56
w/o AoA clustering	85.6	12.2	3.77	3.77	2.84	2.84
w/o Wiener filtering	64.2	13.5	2.89	2.89	1.58	1.58

**Table 3:** Processing latency per module.

Module	Complexity	Latency (ms)	%
Range-FFT	$O(N_{r}\log N_{r}\cdot N_{t})$	66.9	1.6
MTI filtering	$O(N_{r}\cdot N_{t})$	23.6	0.5
AoA estimation	$O(N_{\mathrm{ang}}\cdot N_{\mathrm{virt}}\cdot N_{r}\cdot N_{t})$	4044.0	93.9
3D-DBSCAN	O(|P|log|P|)	72.6	1.7
Wiener filtering	$O(N_{t}\log N_{t})$	101.7	2.4
Total		4308.8	100

## Data Availability

The data supporting the findings of this study are available from the corresponding author upon reasonable request. The data are not publicly available due to privacy and ethical restrictions related to physiological recordings from human participants.
